# Induced pluripotent stem cell macrophages present antigen to proinsulin-specific T cell receptors from donor-matched islet-infiltrating T cells in type 1 diabetes

**DOI:** 10.1007/s00125-019-04988-6

**Published:** 2019-09-12

**Authors:** Kriti Joshi, Colleen Elso, Ali Motazedian, Tanya Labonne, Jacqueline V. Schiesser, Fergus Cameron, Stuart I. Mannering, Andrew G. Elefanty, Edouard G. Stanley

**Affiliations:** 1 Murdoch Children’s Research Institute, Flemington Road, Parkville, VIC 3052 Australia; 2 The Royal Children’s Hospital, Parkville, VIC Australia; 3 Present Address: Sanjay Gandhi Post Graduate Institute of Medical Sciences, Lucknow, India; 4 Immunology and Diabetes Unit, St Vincent’s Institute for Medical Research, Fitzroy, VIC Australia; 5 Department of Paediatrics, University of Melbourne, Parkville, VIC Australia; 6 Department of Anatomy and Developmental Biology, Monash University, Clayton, VIC Australia

**Keywords:** APC generation in vitro, C-peptide, *HLA-DQ8*, iPSCs, Macrophages, Proinsulin, TCRs, Type 1 diabetes

## Abstract

**Aims/hypothesis:**

Type 1 diabetes is an autoimmune disorder characterised by loss of insulin-producing beta cells of the pancreas. Progress in understanding the cellular and molecular mechanisms underlying the human disease has been hampered by a dearth of appropriate human experimental models. We previously reported the characterisation of islet-infiltrating CD4^+^ T cells from a deceased organ donor who had type 1 diabetes.

**Methods:**

Induced pluripotent stem cell (iPSC) lines derived from the above donor were differentiated into CD14^+^ macrophages and tested for their capacity to present antigen to T cell receptors (TCRs) derived from islet-infiltrating CD4^+^ T cells from the same donor.

**Results:**

The iPSC macrophages displayed typical macrophage morphology, surface markers (CD14, CD86, CD16 and CD11b) and were phagocytic. In response to IFNγ treatment, iPSC macrophages upregulated expression of HLA class II, a characteristic that correlated with their capacity to present epitopes derived from proinsulin C-peptide to a T cell line expressing TCRs derived from islet-infiltrating CD4^+^ T cells of the original donor. T cell activation was specifically blocked by anti-HLA-DQ antibodies but not by antibodies directed against HLA-DR.

**Conclusions/interpretation:**

This study provides a proof of principle for the use of iPSC-derived immune cells for modelling key cellular interactions in human type 1 diabetes.

**Electronic supplementary material:**

The online version of this article (10.1007/s00125-019-04988-6) contains peer-reviewed but unedited supplementary material, which is available to authorised users.

## Introduction

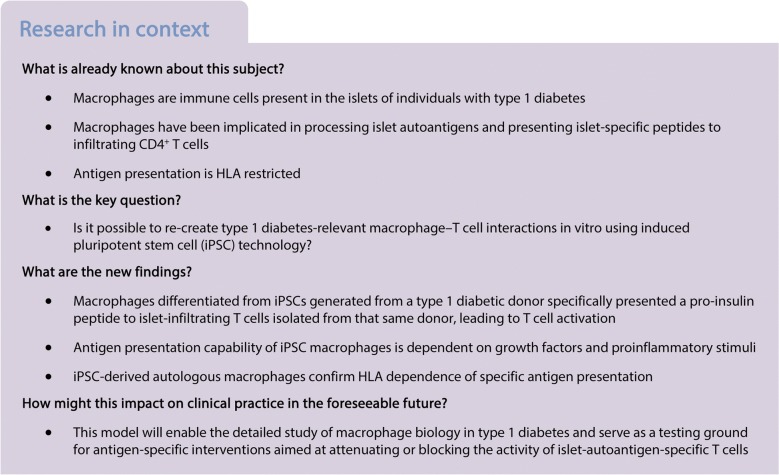



Type 1 diabetes is a chronic autoimmune disorder characterised by T cell-mediated destruction of pancreatic beta cells. A varying preclinical phase of islet inflammation and gradual (and possibly fluctuating) beta cell destruction precedes the onset of clinical symptoms [1], a reality that has meant that studying the pathogenesis of human type 1 diabetes has been challenging.

Studies on human pancreases, such as the Network for Pancreatic Organ donors with Diabetes (nPOD) and the Diabetes Virus Detection Study (DiViD), have shed much-needed light on the human disease process. However, these resources are still limited by the scarcity of pancreatic tissue samples from affected individuals [2, 3]. The retroperitoneal nature of the organ and the inherent risk of pancreatitis make pancreatic biopsies an uncommon procedure and, therefore, most available human data are from the analysis of post-mortem tissue. Furthermore, the long preclinical phase of the disease means that affected individuals only present relatively late in the disease process, making the study of early-disease pathogenesis difficult. Therefore, until recently, most of the current knowledge about disease pathogenesis was extrapolated from rodent models of type 1 diabetes, with the NOD mouse being most commonly used. Whilst the NOD mouse has been pivotal in elucidating certain key pathogenic disease features, there are important differences in the disease patterns between human type 1 diabetes and the NOD mouse [4]. More recently, pluripotent stem cells (PSCs) have been employed to study diabetes caused by mutations in genes that affect beta cell development and function. However, the use of this system to investigate autoimmune responses in type 1 diabetes has been limited [5].

We previously characterised proinsulin-specific islet-infiltrating CD4^+^ T cells isolated from a type 1 diabetic donor [6]. In this study, we tested whether it was possible to use iPSCs derived from this same donor to generate HLA-matched macrophages that could functionally interact with autologous islet-infiltrating T cells.

## Methods

For detailed Methods, please refer to the electronic supplementary materials (ESM).

### Ethical approval

Use of tissue donor material was approved by the St Vincent’s Hospital Human Research Ethics Committee (approval no. SVH HREC-A 011/04). iPSC generation, growth and differentiation were approved by the Royal Children’s Hospital human research ethics committee (approval no. 33001A).

### iPSC generation

iPSC were generated from cryopreserved peripheral blood mononuclear cells (PBMCs) from a type 1 diabetic donor, using a previously described method [7]. Two resulting iPSC lines (‘AF1’ and ‘AF2’) were expanded and characterised further (see ESM Methods for details).

### Immunofluorescence staining

Undifferentiated iPSCs were examined for expression of sex determining region Y-box 2 (SOX2), octamer-binding protein 4 (OCT4) and E-cadherin (ECAD) using immunofluorescence analysis, as detailed in the ESM Methods (see ESM Table 1 for antibody details).

### Generation of iPSC macrophages

iPSCs were differentiated towards the monocyte/macrophage lineage using a protocol based on that reported by Yanagimachi et al. [8]. Macrophage maturation was performed using macrophage colony stimulating factor (M-CSF) or granulocyte monocyte colony stimulating factor (GM-CSF), as specified, following which macrophages were activated using IFN*y* prior to their use in T cell assays (see ESM Methods for further details).

### Macrophage characterisation, T-cell lines and clones and antigen presentation assay

Macrophages were characterised by flow cytometry using antibodies against established surface markers (ESM Table 1) and using May–Grünwald–Giemsa-stained cytospin preparations. Phagocytic activity was determined using a flow cytometry-based fluorescent bioparticle uptake assay (pHrodo Red *Escherichia coli* BioParticles [ThermoFisher, catalogue no. P35361] or carboxyfluorescein succinimidyl ester (CFSE)-labelled *E. coli*; see ESM Methods), whilst functionality was established using antigen presentation assays, as previously described [9].

Isolation of islet-infiltrating T cells underpinning this study was conducted as previously described [6]. In order to facilitate analysis of T cell receptor (TCR) engagement, sequences encoding TCRs from the CD4^+^ T cell clone A1.9 were cloned into the pRRLSIN lentiviral expression vector (pRRLSIN.cPPT.PGK-GFP.WPRE [Addgene; catalogue no. 12252; http://n2t.net/addgene:12252; RRID: Addgene_12,252;www.addgene.org] was a gift from D. Trono) and transduced into a derivative of the T cell leukaemia line SKW3. The resultant line is herewith referred to as SKW3 A1.9 TCR T cells (see ESM Methods for further details). SKW3 A1.9 TCR T cells/CD4^+^ T cells were incubated with iPSC macrophages at a 1:1 ratio and with a synthetic proinsulin peptide (herewith referred to as ‘peptide 11’), C-peptide or islet extract for 24 h. The sequence of C-peptide is EAEDLQVGQVELGGGPGAGSLQPLALEGSLQ, and the sequence of peptide 11 is LQVGQVELGGGPGAGSLQ. The minimum epitope recognised by the TCR from clone A1.9 is VELGGGPGA (see ESM Methods).

T cell activation as a response to antigen presentation was quantified by flow cytometric analysis of CD69 expression. Co-culture of T cell lines with an HLA-matched Epstein-Barr Virus transformed lymphoblastoid B cell line (EBV-BLL) was used as a positive control. Further details are provided in ESM Methods, as well as details of the antigens used.

HLA restriction was determined using blocking monoclonal antibodies against HLA-DR and HLA-DQ (clone L243 and clone SPV-L3, respectively; www.wehi.edu.au/about-structure/laboratory-operations/antibody-services), and by employing antigen presenting cells (APCs) with a known mismatch at the HLA class II loci as a negative control (from iPSC line PB001, from a healthy donor [*HLA-DQ8*^−^]) (see ESM for details).

### CFSE proliferation experiments

CFSE proliferation assays were performed as previously described [10] and as detailed in the ESM Methods. Briefly, CD4^+^ T cells were labelled with CFSE and a subpopulation of uniformly CFSE-labelled cells were isolated by FACS. They were then cultured with iPSC macrophages with or without antigen for 4 days and then analysed for retention of CFSE labelling by flow cytometry.

### Statistics

Data are expressed as mean ± SD. Statistical significance tests included two-sided Student’s *t* tests for paired analyses.

## Results

### Generation and characterisation of iPSC macrophages

**Fig. 1:**
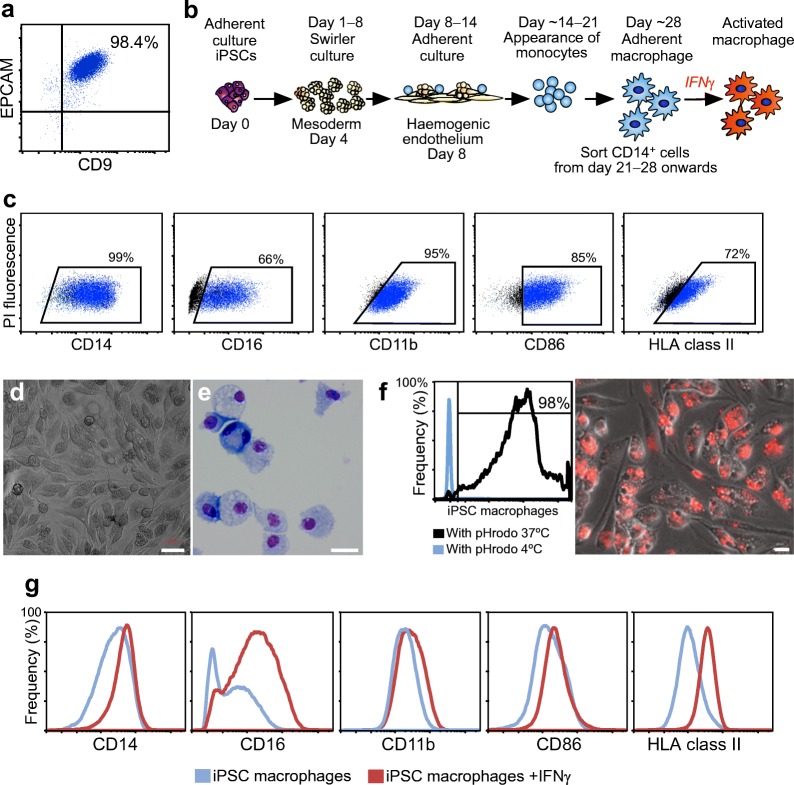
Generation and characterisation of iPSCs and macrophages. (**a**) Flow cytometry analysis of iPSC clone AF1 indicating robust and uniform co-expression of stem cell markers epithelial cell adhesion molecule (EPCAM) and CD9. (**b**) Schematic depicting the differentiation protocol used to generate CD14^+^ monocytes and macrophages (growth factors and cytokine details provided in the ESM methods). (**c**) Flow cytometry analysis of M-CSF-matured iPSC macrophages showing expression of CD14, CD16, CD11b, CD86 and HLA class II. (**d**, **e**). Bright-field images of M-CSF-matured macrophage cultures (**d**) and May–Grünwald–Giemsa-stained cytospin analysis (**e**) showing M-CSF-matured iPSC macrophages have a typical macrophage morphology. Scale bars, 50 μm (**d**) and 100 μm (**e**). **(f**) Flow cytometry analysis of M-CSF-matured iPSC macrophages following incubation with pHrodo Red *E. coli* BioParticles at 4°C and 37°C, showing bead uptake at 37°C. Horizontal and vertical lines within the plot represent the position and width of the pHrodo^+^ population and the pHrodo^−^ population (using pHrodo-*E. coli* at 4°C), respectively. A fluorescent image of M-CSF-matured iPSC macrophages with pHrodo Red *E. coli* BioParticles at 37°C is also presented, showing intracellular fluorescence indicative of phagocytosis. Scale bar, 20 μm. (**g**) Flow cytometry analysis of M-CSF-matured iPSC macrophages showing changes in the level surface marker expression, particularly CD16 and HLA class II, following treatment with IFNγ. PI, propidium iodide

The iPSCs generated in this study possessed PSC-like morphology (ESM Fig. 1a) and demonstrated robust expression of the stem cell surface markers epithelial cell adhesion molecule (EPCAM) and CD9 (Fig. 1a and ESM Fig. 1b; *n* = 1, analysed in duplicate) and transcription factors OCT4 and SOX2 (ESM Fig. 1c). A schematic summarising the protocol used for generating iPSC macrophages is shown in Fig. 1b. Flow cytometric analysis showed that iPSC macrophages expressed typical macrophage markers CD14, CD11b and CD86, with a substantial proportion showing expression of CD16 and HLA class II (Fig. 1c and ESM Fig. 1d; *n* = 1, analysed in duplicate). Under bright field microscopy, iPSC macrophages displayed a uniform morphology, whilst May–Grünwald–Giemsa-stained cytospin preparations revealed large mononuclear cells with the vacuolated cytoplasm typical of macrophages (Fig. 1d, e). iPSC macrophage function was assessed by testing the capacity of these cells to phagocytose fluorescent *E. coli* bioparticles. Flow cytometry analysis showed that iPSC macrophages were highly phagocytic, with >98% of cells showing uptake of *E. coli* bioparticles (Fig. 1f); this phagocytosis was reduced by the actin polymerisation inhibitor cytochalasin D (ESM Fig. 1f; data from two independent experiments using pHrodo-*E. coli* [*n* = 1] and CFSE-*E. coli* [*n* = 1]). Finally, treatment of cultures with IFNγ upregulated the expression of macrophage surface markers, particularly CD16 and HLA class II (AF1, Fig. 1g). This was also confirmed by independent experiments with AF2 (ESM Fig. 1e; *n* = 1) and by three independent experiments performed with AF1 and AF2 (ESM Fig. 1g, *n* = 3). Overall, these findings indicate that iPSC macrophages display typical features of similar cells isolated from in vivo sources, including surface marker expression and basic functional properties.

### Antigen presentation by iPSC macrophages

**Fig. 2:**
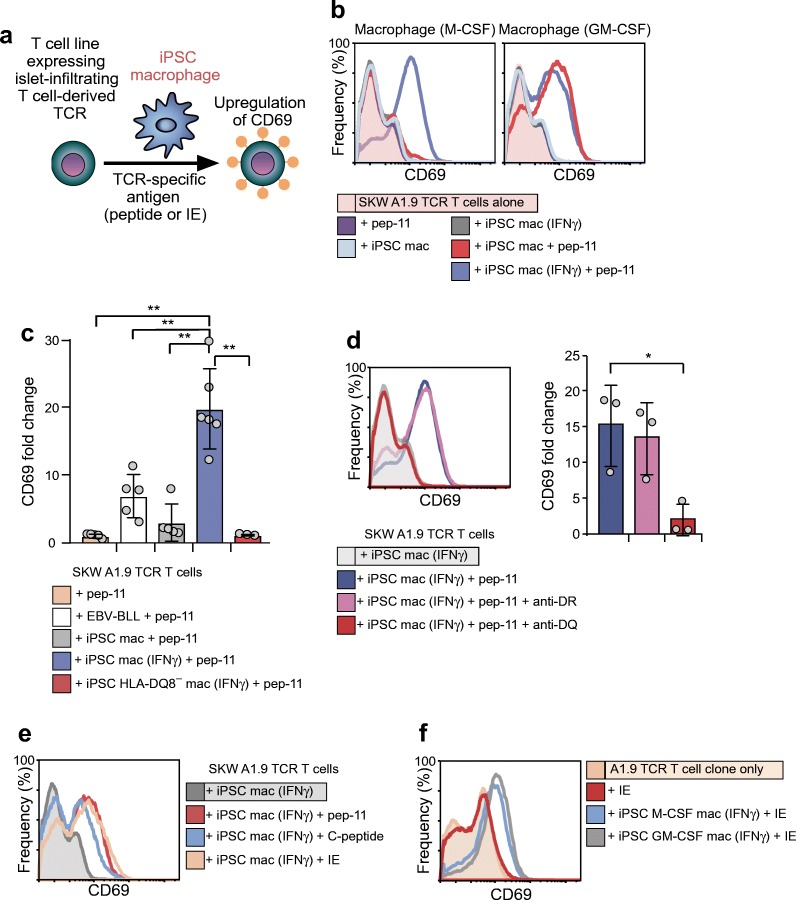
iPSC macrophages present antigen to a TCR from islet-infiltrating T cells of the primary donor. (**a**) Schematic representation of the experimental design showing how co-incubation of T cell lines bearing a specific TCR with iPSC macrophages and TCR-specific antigen leads to upregulation of the T cell activation marker CD69. (**b**) Representative flow cytometry histograms showing that presentation of a TCR-specific C-peptide-derived peptide (referred to as ‘peptide 11’) is dependent on prior IFNγ treatment of M-CSF-, but not of GM-CSF-matured macrophages (*n* = 1). (**c**) Peptide-presenting capacity of M-CSF-matured iPSC macrophages compared with an *HLA-DQ8*-expressing EBV-BLL and macrophages derived from the PB001 line (*HLA-DQ8*^−^). The *y*-axis values are given as fold change in the mean fluorescence intensity of CD69 labelling relative to that obtained for IFNγ-treated iPSC macrophages/EBV-BLL in the absence of antigen (peptide 11). Data are shown as the mean ± SD for three or more independent experiments. **p < 0.01, by two tailed unpaired *t* test. (**d**) Representative flow cytometry histograms showing that antigen presentation by IFNγ-treated M-CSF-matured macrophages can be blocked by anti-HLA-DQ antibodies but not anti-HLA-DR antibodies. Histogram plot summarising the blocking of antigen presentation by anti-HLA-DQ but not by anti-HLA-DR antibodies. The *y*-axis values are given as fold change in the mean fluorescence intensity of CD69 labelling relative to that obtained for IFNγ-treated GM- and M-CSF matured iPSC macrophages in the absence of antigen (peptide 11). Data are shown as the mean ± SD for three independent experiments. *p < 0.05, by two tailed unpaired Student’s *t* test. (**e**) Flow cytometry analysis showing upregulation of CD69 on the SKW A1.9 TCR T cells in response to the specified antigen presented by IFNγ-treated M-CSF-matured iPSC macrophages (*n* = 1). (**f**) Flow cytometry analysis showing upregulation of CD69 on the islet derived A1.9 T cell clone in response to islet extract processed and presented by iPSC macrophages matured with either M-CSF or GM-CSF (*n* = 1). anti-DR, anti-HLA-DR antibodies; anti-DQ, anti-HLA-DQ antibodies, IE, islet extract; mac, macrophages; pep-11, peptide 11

To assess the antigen-presenting capabilities of iPSC macrophages, we used a flow cytometric assay in which macrophages were co-cultured with T cell lines expressing a proinsulin-specific TCR derived from a previously characterised islet-infiltrating CD4^+^ T cell clone, A1.9 (SKW3 A1.9 TCR T cells) [6]. T cell activation was assessed by measuring antigen-dependent upregulation of CD69, an antigen-specific early T cell activation marker (Fig. 2a). Flow cytometry indicated that CD69 expression was upregulated in SKW3 A1.9 TCR T cells when co-cultured with iPSC macrophages in the presence of a TCR-specific peptide antigen (‘peptide 11’; Fig. 2b). In the case of iPSC macrophages matured with M-CSF, effective antigen presentation required prior treatment with IFNγ, whilst those matured with GM-CSF were able to present peptide without further activation (Fig. 2b,c), a difference that may relate to HLA class II expression (ESM Fig. 2a).

We also compared the antigen-presenting capacity of iPSC macrophages with *HLA-DQ8*-expressing EBV-BLL cells, as well as HLA class II mismatched iPSC macrophages generated from a stock iPSC line (Fig. 2c). In these experiments, CD69 induction was measured as fold change over CD69 expression of T cell–APC cultures in which antigenic peptide was absent. These experiments confirmed that IFNγ-treated iPSC macrophages derived from the syngeneic donor were effective at presenting antigen and were more potent than the EBV-BLL cells, whilst iPSC macrophages from an *HLA-DQ8* negative donor failed to induce CD69 expression on SKW3 A1.9 TCR T cells in response to peptide 11 (also see ESM Fig. 2b, c).

We compared the induction of CD69 on target T cells in the absence or presence of HLA blocking antibodies directed against HLA-DR and HLA-DQ. We showed that HLA-DR blocking antibodies did not affect the level of CD69 induction on SKW3 A1.9 TCR T cells, whilst HLA-DQ blocking antibodies significantly reduced CD69 upregulation (Fig. 2d). These findings confirm our previous results indicating that activation of the A1.9 TCR is HLA-DQ dependent [6].

In order to examine whether iPSC macrophages had mature phagocytic and endocytic functionality, we tested their capacity to process heterogeneous protein mixtures, represented by islet cell extract [11], as well as full length C-peptide, and to activate SKW3 A1.9 TCR T cells. The findings indicated that iPSC macrophages could induce a similar level of CD69 upregulation in target T cells when provided with peptide 11, C-peptide or islet extract (Fig. 2e). This result was confirmed by examining the ability of iPSC macrophages to activate the primary CD4^+^ T cell clone A1.9 from which the A1.9 TCR was isolated. In this setting, iPSC macrophages, matured with either M-CSF or GM-CSF, processed and presented antigen to the A1.9 primary T cell clone, measured by CD69 upregulation (Fig. 2f). As expected, we also observed that the A1.9 CD4^+^ cell clone could efficiently present peptide 11, leading to its self-activation (ESM Fig. 2d). By contrast, self-activation was not observed in cultures provided with islet extract, presumably reflecting the inability of the T cells to take up and process complex mixtures (Fig. 2f). Self-activation did not lead to a profound proliferative response, as measured by CFSE dilution, whereas activation driven by iPSCs macrophages in the presence of antigen was predictably far more robust (ESM Fig. 2e). Tellingly, self-activation was not observed using the SKW3 A1.9 TCR T cell lines, which lack the appropriate HLA class II for antigen presentation, underlining the value of using genetically modified T cell lines for examining APC–T cell interactions.

## Discussion

Our study is the first to report the generation of iPSC-derived APCs from a type 1 diabetes donor and to examine their functionality using autologous, islet-infiltrating T cells. We found, whilst M-CSF-matured iPSC macrophages required IFNγ treatment to induce upregulation of HLA class II and present antigen, those matured with GM-CSF displayed a more activated phenotype, underlined by their capacity to present antigen in the absence of IFNγ. M-CSF is thought to play a role in the homeostatic maintenance of monocyte and macrophage populations, whereas GM-CSF is produced predominantly under inflammatory conditions [12]. In this context, it is tempting to speculate that GM-CSF-matured macrophages could mimic the macrophage biology in an autoimmune condition, like type 1 diabetes. Similarly, local production of IFNγ by activated T cells may also serve to prime macrophages for participation in a feedback loop in which islet-resident APCs phagocytose beta cell debris and present autoantigens to infiltrating T cells.

The use of donor-matched iPSC-derived APCs for the analysis of T cell responses has three major advantages over non-autologous HLA-matched APCs (such as the EBV-BLL cells used as a control in this study). First, prior detailed knowledge of the HLA genotype of a particular donor is not required to initiate a search for potential antigens. Second, as seen in this study, iPSC macrophages produce a lower level of background activation compared with that induced by control APCs, potentially enabling lower levels of T cell activation to be detected. This model may also have the capacity to incorporate individual differences in APC function that may contribute to the pathogenesis of type 1 diabetes. Finally, the ability to genetically modify iPSCs presents the opportunity to probe the genetics of the antigen presentation process itself, opening up new avenues of investigation into the role of specific genes in the cellular interactions that underlie type 1 diabetes.

## Electronic supplementary material

ESM (PDF 1370 kb)

## Data Availability

The datasets generated and/or analysed during the current study are available from the corresponding author upon reasonable request. Resources, including iPSC lines, generated and/or analysed during the current study are available from the corresponding author upon reasonable request.

## Abbreviations

APC: Antigen presenting cell
CFSE: Carboxyfluorescein succinimidyl ester
EBV-BLL: Epstein-Barr Virus transformed lymphoblastoid B cell line
GM-CSF: Granulocyte monocyte colony stimulating factor
iPSC: Induced pluripotent stem cell
M-CSF: Macrophage colony stimulating factor
OCT4: Octamer-binding protein 4
PSC: Pluripotent stem cell
SOX2: Sex determining region Y-box 2
TCR: T cell receptor
