# Supplementary material for: Induced pluripotent stem cell macrophages present antigen to proinsulin-specific T cell receptors from donor-matched islet-infiltrating T cells in type 1 diabetes
Source: Diabetologia. 2019 Sep 12;62(12):2245–51. doi: 10.1007/s00125-019-04988-6 (PMC6861360; doi:10.1007/s00125-019-04988-6)
Supplement: Supplementary file 1 — (PDF 1370 kb) [file 125_2019_4988_MOESM1_ESM.pdf]

Supplementary Material for

iPSC Macrophages present antigen to pro-insulin specific T-cell receptors from donor matched islet infiltrating T-cells in type 1 diabetes

Kriti Joshi[1,2,6], Colleen Elso[5], Ali Motazedian[1,3], Tanya Labonne[1], Jacqueline V. Schiesser[1,3], Fergus Cameron[1,2,3], Stuart I. Mannering[5,#], Andrew G. Elefanty[1,3,4,#] and Edouard G Stanley[1,3,4 #,*]

[1]Murdoch Children's Research Institute, Flemington Road, Parkville, 3052, Australia.

[2]The Royal Children's Hospital, Flemington Road, Parkville, 3052, Australia.

[3]Department of Paediatrics, University of Melbourne, Parkville, Victoria, 3050, Australia

[4]Department of Anatomy and Developmental Biology, Monash University, Clayton, Victoria, 3800, Australia.

[5]Immunology of Diabetes Unit, St. Vincent's Institute for Medical Research, Fitzroy, Victoria, 3065, Australia

[6]Current address, Sanjay Gandhi Post Graduate Institute of Medical Sciences, Lucknow, 226014, India.

# Equal contributors

* To whom correspondence should be addressed:

This File contains, in the following order:

1. Supplementary Methods and Materials
2. References relevant to supplementary materials
3. Supplementary Table 1
4. Supplementary Figures 1 and 2, and accompanying legends

Materials and Methods

iPSC Generation: We generated iPSCs from cryopreserved PBMCs isolated from a tissue donor with type 1 diabetes and from whom we had previously isolated islet-infiltrating T-cells. The characteristics of the Type 1 diabetic donor have been previously described [1]. In brief this donor developed T1D at the age of 16 and died during an episode of hypoglycaemia, 3 years after diagnosis. He was positive for GAD-65 and IA-2 antibodies and carried a high risk HLA genotype; HLA-A*01:01, 02:01; B*08:01, 51:01; DRB1*03:01, 04:04; DQA1*03:01, DQB1*03:02; DQA1*05:01, DQB1*02:01.

In order to generate iPSCs, donor derived PBMCs were cultured for 7 days in StemSpan™ SFEM II (STEMCELL Technologies, (https://www.stemcell.com) containing StemSpan™ erythroid expansion supplement (STEMCELL Technologies). Erythroid progenitors were reprogrammed using a Cytotune™-iPS 2.0 Sendai Reprograming kit (ThermoFisher Scientific). Transduced cells were plated on culture dishes seeded with irradiated mouse embryonic fibroblasts (MEFs), and maintained in human pluripotent stem cell medium containing Knockout DMEM/20%Knockout serum replacer supplemented with 50ng/mL of FGF2 [2]. Two resulting iPSC lines (AF1 and AF2) were expanded and assessed for the expression of stem cell markers using flow cytometry and subjected to karyotype analysis using the Infinium CoreExome-24 SNP arrays. Cell lines were adapted to feeder free conditions prior to initiating differentiation and were maintained in Essential 8™ Medium (ThermoFisher Scientific).

Immunofluorescence staining: Undifferentiated iPSCs were grown on glass coverslips. The coverslips were washed with 1 x PBS then fixed in 4% PFA for 10 minutes at room temperature. Following this, the coverslips were placed in blocking buffer (10% FBS + 0.1% Triton-X in 1x PBS) for 1 hour at room temperature. Primary antibodies (see ESM table 1 for catalogue numbers and dilutions) were diluted in blocking buffer and incubated overnight at 4ºC. Secondary antibodies (ESM table 1) were diluted in blocking buffer and incubated at room temperature for 1 hour. Fluoromount-G (ThermoFisher Scientific) was applied to the coverslips which were then mounted on slides. Slides were imaged using a Zeiss confocal LSM 780 inverted microscope (https://www.zeiss.com). Image analysis was performed using ImageJ software, Version 1.0, 1-6-2019 to 30-7-2019, https://imagej.net).

Generation of iPSC Macrophages: Differentiation towards the monocyte macrophage lineage was essentially performed as described [3], with the variation that embryoid bodies were formed using rotational cultures [4]. Embryoid bodies were formed in media supplemented with 20 ng/ml recombinant human (rh) bone morphogenetic protein 4 (BMP4, R&D Systems, R&D systems, https://www.rndsystems.com), 40 ng/ml rh vascular endothelial growth factor (VEGF, PeproTech, https://www.peprotech.com) 40 ng/ml rh stem cell factor (SCF, PeproTech), 10 ng/ml rh Fibroblast growth factor 2 (FGF2 5ng/ml used for the first day) (PeproTech), 5 ng/nl rh activin A (R&D Systems) day and CHIR99021 0.2 µM (Tocris Biosciences, https://www.tocris.com)(set up medium). Media was changed on day 1 with set up medium lacking Activin A, and again on day 2, with set up medium lacking both Activin A and CHIR. After day 8, EBs were transferred onto matrigel-coated, 6-well plates at ~ 20 EBs/well in media supplemented with 20 ng/ml rh VEGF and 20 ng/ml SCF, 10 ng/ml FGF2, 25 ng/ml of rh interleukin-3 (IL-3) (Peprotech) and 50 ng/ml of macrophage colony stimulating factor (M-CSF) (Peprotech). 50 ng/ml rh FLT3 receptor ligand (FLT3L, PeproTech) and 25 ng/ml granulocyte monocyte colony stimulating factor (GM-CSF) was added to the above media from day 11 of culture. The appearance of monocytic cells was assessed by flow cytometric analysis for CD14 expression at differentiation day 14. Monocytic cells shed from the EBs were further matured in media supplemented with 25 ng/ml of IL-3, 50 ng/ml of M-CSF 50 ng/ml FLT3L and 25 ng/ml GM-CSF. Post CD14 sorting, $CD14^{+\,high}$ cells of monocytic lineage were matured for 5-7 days

in media containing 100 ng/ml of M-CSF (or 50 ng/ml of GM-CSF when specified) to yield macrophages, which were subsequently activated with 20ng/ml of IFNγ prior to use in T cell assays.

Flow cytometry and cell sorting: Adherent macrophage cultures were detached using TrypLE select (Thermofisher) to yield single cell suspension. Cells were washed with PBS and then resuspended in 100 μl of FACS wash (PBS with 2% Fetal calf serum). Cells were labelled with fluorophore-conjugated antibodies at the dilutions specified in Supplementary Table 2 and the mixture incubated for 20 minutes on ice. The cell suspension was washed twice with FACS wash to remove unbound antibodies and resuspended in FACS wash containing 1 μg/ml propidium iodide. Flow cytometric analysis was performed using a BD Fortessa flow cytometer. Flow cytometry gates were set relative to samples labelled with an isotype control antibody. Cell sorting was performed using a FACS Aria cell sorter (BD BioSciences, https://www.bdbiosciences.com). See ESM Table 1 for antibodies used for flow cytometric analysis.

Characterisation of iPSC macrophages: For flow cytometric analysis, adherent macrophage cultures were first detached using TrypLE select (ThermoFisher) to yield a single cell suspension. Labelling of cells for flow cytometry was performed as detailed above using the fluorophore-conjugated antibodies specified in Supplementary Table 1.

Characterisation of iPSC macrophages using Giemsa-May Grunwald staining: Cells were collected in PBS and centrifuged at 900rpm for 5 minutes using a Thermo Shandon Cytospin 4 (https://www.thermofisher.com). Slides were air dried for 30 minutes, stained with 50% May-Grünwald solution for 5 min, washed twice with distilled water, then stained with 5% Giemsa for 20 minutes and finally rinsed with distilled water. Slides were air dried overnight and mounted with cover slips using DPX. Images were taken using Zeiss Axiocam mounted on an Axiovert 200 inverted microscope.

Characterisation of iPSC macrophages with phagocytosis assays: For main figure 1f, iPSC Macrophages were plated at 100,000 cells/well of a 12 well plate and incubated overnight at 37ºC to allow cells to adhere. pHrodo^{TM} Red *E. coli* BioParticles$^{®}$ Conjugate (ThermoFisher, cat # P35361), reconstituted to a concentration of 1mg/ml, were added to macrophage cultures and incubated for 1-2 hours at 37˚C. Cells were then harvested using TrypLE select (ThermoFisher) and analysed using a BD Fortessa flow cytometer (https://www.bdbiosciences.com). A separate sample of cells with the added bioparticles was kept on ice as a negative control. For ESM figure 1g, cells from late stage (week 3) GM-CSF and M-CSF supplemented macrophage cultures were harvested using TrypLE select and collected by centrifugation. Cells were resuspended in medium containing either CFSE-(See below) or pHRodo-labelled (ThermoFisher, cat # P35361) E.coli bioparticles in the presence or absence of 20 μM Cytochalasin D (Sigma, cat# C2618, (https://www.sigmaaldrich.com). Cells were incubated at 37ºC for 1 hour with mixing. Additional controls included cells incubated in the presence of bioparticles at 4ºC. Unbound particles were removed by washing cells 4 times with 4 mls of FACS wash (PBS with 2% Fetal Calf Serum). After the final wash, cell pellets were resuspended in FACS wash containing a PE-Cy7 conjugated anti-CD16 antibody (ESM table 1) and processed for flow cytometry as described below.

CFSE labelling of E.coli. CFSE labelled E.coli were generated by incubating a 10μl pellet of heat killed (65ºC, 15 minutes) DH10β E.coli in 1 μM CFSE (resuspended in PBS) at 37º for 10 minutes. Unbound CFSE was removed by washing 5 times with 1 ml of PBS. Labelled E.coli was resuspended in 1 ml of PBS prior to use

T-cell line culture. The SKW3 T-cell line used in this study was maintained in RPMI 1640 supplemented with 10% FCS.

Antigen presentation assays: T-cell activation assays were performed essentially as described previously[5]. Briefly, SKW3 A1.9 TCR T cells / CD4+ T cells were incubated with 20-30,000 iPSC Macrophages at a 1:1 ratio and the specified concentrations of a synthetic proinsulin peptide, c-peptide or islet extract for 24 hours. T cell activation as a response to antigen presentation was quantified by flow cytometric analysis of CD69 expression, an antigen specific early T cell activation marker [6]. Co-culture of T cell lines with an HLA-matched EBV transformed B lymphoblastoid line (EBV BLL) (KJ; HLA-DRB1*03:01, 04:04; DQB1*02:01, 03:02) was used as a positive control. HLA blocking experiments were performed as described below.

Synthetic proinsulin peptides, c peptide and islet extract: Synthetic Peptide 11 was synthesized by Purar Chemicals (Shanghai, China) and reconstituted in 40% acetonitrile, 0.5% acetic acid, and water to 5 mmol/L, aliquoted, and stored at -80°C. Full length C peptide ($PI_{33–63}$) was purchased from Abcam. Islet lysates were prepared as previously described [7]. The sequence of C-peptide is **EAEDLQVGQVELGGGPGAGSLQPLALEGSLQ,** and the sequence of Peptide-11 is highlighted in yellow. The minimum epitope recognized by the TCR from clone A1.9 is shown in red text.

HLA specificity analysis: HLA restriction was determined in two ways. First, we tested the effect of blocking HLA engagement using monoclonal antibodies specific for HLA-DR (clone L243), and -DQ (clone SPV-L3), which were added to cultures at a final concentration of 5–10 µg/mL. We also generated macrophages from an iPSC line (PB001) from healthy donor which was not HLA matched to the T1DM donor [8] derived TCRs. These HLA mismatched iPSC macrophages were included as a negative control for subsequent T cell activation assays.

Statistics: All experiments were analysed using Microsoft Excel version 16.27 (Microsoft Corporation, www.microsoft.com) and GraphPad Software Inc, Prism 7 and Prism 8 (www.graphpad.com)

Statistical significance tests included two-sided Student's t-tests for paired analyses.

References relevant to supplementary materials:

[1] Pathiraja V, Kuehlich JP, Campbell PD, et al. (2015) Proinsulin-specific, HLA-DQ8, and HLA-DQ8-transdimer-restricted CD4+ T cells infiltrate islets in type 1 diabetes. Diabetes 64(1): 172-182. 10.2337/db14-0858
[2] Costa M, Sourris K, Hatzistavrou T, Elefanty AG, Stanley EG (2008) Expansion of human embryonic stem cells in vitro. Curr Protoc Stem Cell Biol Chapter 1: Unit 1C 1 1-1C 1 7. 10.1002/9780470151808.sc01c01s5
[3] Yanagimachi MD, Niwa A, Tanaka T, et al. (2013) Robust and highly-efficient differentiation of functional monocytic cells from human pluripotent stem cells under serum- and feeder cell-free conditions. PLoS One 8(4): e59243. 10.1371/journal.pone.0059243
[4] Carpenedo RL, Sargent CY, McDevitt TC (2007) Rotary suspension culture enhances the efficiency, yield, and homogeneity of embryoid body differentiation. Stem Cells 25(9): 2224-2234. 10.1634/stemcells.2006-0523
[5] Gras S, Chen Z, Miles JJ, et al. (2010) Allelic polymorphism in the T cell receptor and its impact on immune responses. J Exp Med 207(7): 1555-1567. 10.1084/jem.20100603
[6] Simms PE, Ellis TM (1996) Utility of flow cytometric detection of CD69 expression as a rapid method for determining poly- and oligoclonal lymphocyte activation. Clin Diagn Lab Immunol 3(3): 301-304
[7] So M, Elso CM, Tresoldi E, et al. (2018) Proinsulin C-peptide is an autoantigen in people with type 1 diabetes. Proc Natl Acad Sci U S A 115(42): 10732-10737. 10.1073/pnas.1809208115
[8] Vlahos K, Sourris K, Mayberry R, et al. (2018) Generation of iPSC lines from peripheral blood mononuclear cells from 5 healthy adults. Stem Cell Res 34: 101380. 10.1016/j.scr.2018.101380

Supplementary table 1: details of antibodies used in this study.

Antibody	Company	Cat number	Clone	RRID	Method	Dilution
EPCAM APC	Biolegend	324208	9C4	AB_756082	Flow cytometry	1:100
CD9-FITC	BD Pharmingen	555371	M-L13	AB_395773	Flow cytometry	1:30
CD 14 PECy7	Biolegend	301814	M5E2	AB_389353	Flow cytometry	1:50
CD 11b FITC	BD Pharmingen	557701	ICRF44	AB_2129268	Flow cytometry	1:50
CD 16 PECy7	BioLegend	302016	3G8	AB_314216	Flow cytometry	1:50
CD 86 APC	BioLegend	305412	IT2.2	AB_493231	Flow cytometry	1:100
HLA DR FITC	BD Pharmingen	347363	L243	AB_400291	Flow cytometry	1:30
CD 69 APC	BioLegend	310909	FN50	AB_314844	Flow cytometry	1:50
OCT4	Santa Cruz	sc-5279	C-10	AB_628051	Immunofluorescence	1:200
SOX2	R&D systems	MAB2018	245610	AB_358009	Immunofluorescence	1:200
ECAD	R&D systems	AF648	Polyclonal	AB_355504	Immunofluorescence	1:250
Donkey anti-mouse 594	Invitrogen	A-21203	Polyclonal	AB_141633	Immunofluorescence	1:1000
Donkey anti-goat 488	Invitrogen	A-11055	Polyclonal	AB_2534102	Immunofluorescence	1:1000

Biolegend (https://www.biolegend.com), BD Pharmingen (https://www.bdbiosciences.com), Santa Cruz (https://www.scbt.com), R&D systems, (https://www.rndsystems.com), Invitrogen (https://www.thermofisher.com),

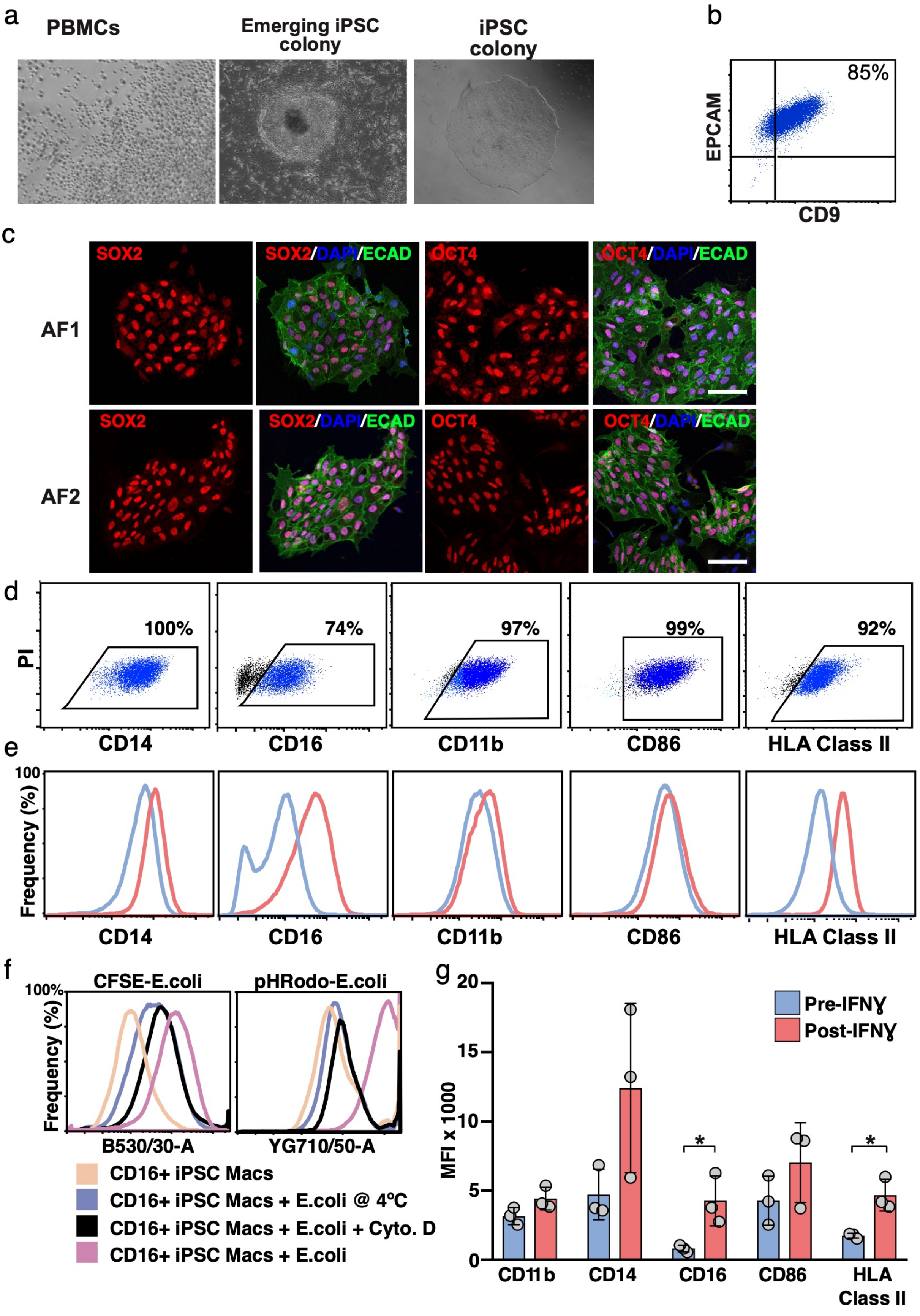
a
PBMCs
Emerging iPSC colony
iPSC colony
b
85%
EPCAM
CD9
c
SOX2
SOX2/DAPI/ECAD
OCT4
OCT4/DAPI/ECAD
AF1
AF2
d
PI
100%
74%
97%
99%
92%
CD14
CD16
CD11b
CD86
HLA Class II
e
100
Frequency (%)
CD14
CD16
CD11b
CD86
HLA Class II
f
CFSE-E.coli
pHRodo-E.coli
100%
Frequency (%)
B530/30-A
YG710/50-A
CD16+ iPSC Macs
CD16+ iPSC Macs + E.coli @ 4ºC
CD16+ iPSC Macs + E.coli + Cyto. D
CD16+ iPSC Macs + E.coli
g
MFI x 1000
20
15
10
5
0
Pre-IFNϒ
Post-IFNϒ
*
*
CD11b
CD14
CD16
CD86
HLA Class II

ESM figure 1. Generation and characterisation of iPSC derived macrophages. a. Bright field image showing donor PBMC's Day1 post reprogramming, appearance of iPSCs colonies 3 weeks post reprogramming and morphology of iPSCs in feeder free culture conditions. **b**. Flow cytometry analysis of iPSC clone AF2 indicating robust co-expression of stem cell markers EPCAM and CD9. **c**. Immunofluorescence images of paraformaldehyde fixed AF1 (upper row) and AF2 iPSC cells (lower row) showing robust labelling of nuclei with antibodies directed against the stem cell associated transcription factors OCT4 and SOX2. Nuclei were identified with DAPI and cell boundaries outlined by labelling with anti-ECAD antibodies. Scale bar is 100 **μ**m. **d.** Flow cytometry analysis of iPSC macrophages differentiated from iPSC lines AF2 for expression of key lineage markers on of CD14, CD16, CD86, CD11b and HLA-Class II. **e**. Flow cytometry analysis of CD14, CD16, CD86, CD11b and HLA-Class II on iPSC macrophages before (**blue line**) and after (red line) stimulation with 20ng/ml IFNγ. **f.** Flow cytometry analysis of fluorescently labelled bioparticle uptake by CD16+ iPSC macrophages under the conditions specified. Note that for the phRodo-E.coli uptake experiment, fluorescence was measured in the far red YG710/50-A channel to compensate for the use of the PE-Cy7-conjugated antibody used to identify CD16+ macrophages. **g.** Histograms summarising results of three independent experiments showing up regulation of CD14, CD16, CD86, HLA Class II and CD11b on M-CSF matured iPSC Macrophages (as increase in mean fluorescence intensity, MFI) after stimulation with 20ng/ml IFNγ. Data is shown as the mean +/- SD for 3 independent experiments. * indicates $P<0.05$ by two tailed unpaired student t-test.

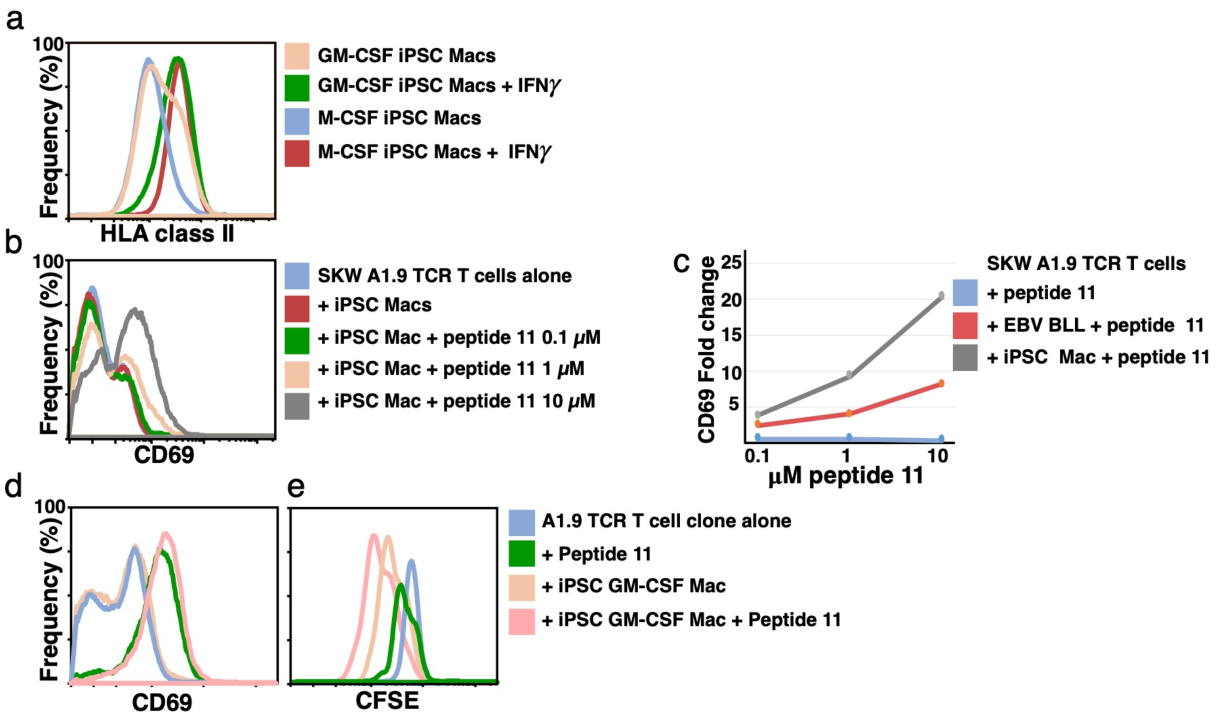

ESM figure 2. Proinsulin derived peptide presentation by iPSC Macrophages, the CD4+ T-cell clone A1.9, T-cell lines bearing the A1.9 TCR and an HLA matched EBV transformed B-lymphoblastoid line (EBV BLL). **a**. Flow cytometry analysis showing the relative level of HLA class II expression in GM-CSF versus M-CSF matured macrophages with and without stimulation with IFNγ. **b.** Flow cytometry histogram overlays showing the effect of increasing peptide 11 concentration on CD69 upregulation on SKW cells expressing the A1.9 TCR co-cultured with M-CSF matured iPSC Macrophages. c. Graphical summary of data derived from a single experiment examining the effect of increasing concentrations of peptide 11 on CD69 upregulation on SKW3 A1.9 TCR T cell line bearing pro-insulin specific TCRs when presented by M-CSF matured iPSC Macrophages as compared to the responses elicited with the use of the HLA matched EBV transformed B-lymphoblastoid line. All iPSC derived macrophages used in this panel were activated with 20ng/ml IFNγ for 48 hours prior to use. **d**. The islet derived CD4+ T-cell clone can self-present the TCR specific peptide (peptide 11) and addition of GM-CSF matured IFNγ treated iPSC Macrophages does not increase the level of T-cell activation over and above that induced by self-presentation. The key identifying each condition is indicated to the right of panel d. **e**. CFSE dilution (proliferation) experiment showing that self-presentation is a relatively weak driver of proliferation and that activated macrophages alone are also capable of eliciting a strong proliferation response (but does not induce CD69 – see, d). The combination of peptide and activated macrophages provides the strongest proliferative stimulus.
